# Does coffee drinking have beneficial effects on bone health of Taiwanese adults? A longitudinal study

**DOI:** 10.1186/s12889-018-6168-0

**Published:** 2018-11-20

**Authors:** Huan-Cheng Chang, Chuan-Fa Hsieh, Yi-Chin Lin, Disline Manli Tantoh, Pei-Chieh Ko, Ya-Yu Kung, Mei-Chi Wang, Shu-Yi Hsu, Yi-Ching Liaw, Yung-Po Liaw

**Affiliations:** 1 Division of Family Medicine, Department of Community Medicine, Landseed Hospital, Taoyuan, Taiwan; 2 Department of Health Care Management, Chang Gung University, Taoyuan, Taiwan; 3 Department of Medical Education and Research, Landseed Hospital, Taoyuan, Taiwan; 4 Center for General Education, Hsin Sheng College of Medical Care and Management, Taoyuan, Taiwan; 5 Institute of Nutritional Science, Chung Shan Medical University, Taichung, Taiwan; 6 Department of Public Health and Institute of Public Health, Chung Shan Medical University, No. 110, Sec. 1 Jianguo N. Rd, Taichung City, 40201 Taiwan; 7 Division of Health Management, Landseed Hospital, Taoyuan, Taiwan; 8 Institute of Clinical Medicine, National Yang-Ming University, Taipei, Taiwan; 9 Department of Family and Community Medicine, Chung Shan Medical University, Taichung, Taiwan

**Keywords:** Coffee, Osteoporosis risk, Sex, Menopausal status, Taiwan

## Abstract

**Background:**

Results from studies investigating the association between coffee consumption and osteoporosis or bone mineral density (BMD) have been inconsistent. This longitudinal study was performed to assess the effect of coffee drinking on bone health of Taiwanese adults.

**Methods:**

Data were retrieved from the Li-Shin (Landseed) Hospital in Taoyuan City. In 2006, 6152 participants completed a questionnaire on coffee drinking and other lifestyle factors. In 2014, 5077 of them were followed up. Nonetheless, a total of 2395 participants with incomplete data were excluded. The final analyses included 2682 participants comprising 1195 men and 1487 women (706 premenopausal and 781 postmenopausal). T-scores were derived from the osteo-sono assessment index (OSI) which is a surrogate of BMD. Coffee drinking was categorized as “no, medium, and high” based on the number of cups that were consumed per week in both 2006 and 2014.

**Results:**

In general, medium and high coffee drinking were associated with higher T-scores. However, significant results were observed only among high drinkers (β = 0.158; *P* = 0.0038). Nonetheless, the test for linear trend was significant (*P* = 0.0046). After stratification by sex, medium and high coffee drinking were associated with higher T-scores. However, significant results were prominent only among high male drinkers (β = 0.237; *P* = 0.0067) and the test for trend was significant (*P* = 0.0161). Based on menopausal status, coffee drinking was associated with higher T-scores. Nevertheless, significant results were found only among premenopausal women (β = 0.233; *P* = 0.0355 and β = 0.234; *P* = 0.0152 for medium and high coffee drinking, respectively. The test for linear trend was significant (*P* = 0.0108).

**Conclusion:**

Coffee drinking was significantly associated with higher T-scores hence, a lower risk of osteoporosis in men and premenopausal women.

**Electronic supplementary material:**

The online version of this article (10.1186/s12889-018-6168-0) contains supplementary material, which is available to authorized users.

## Background

Osteoporosis is a skeletal disorder characterised by reduced bone mass and deteriorated microarchitecture of bone tissue which subsequently lead to increased bone fragility and fractures [1–3]. Low bone mineral density is among the important clinical risk factors for osteoporosis and osteoporotic fractures [1].

Osteoporosis is a serious public health issue. The economic and public health burden of osteoporosis and osteoporotic fractures cannot be underemphasized. In the year 2000, the global annual incidence and prevalence of osteoporotic fractures were estimated at 8.9 and 56 million, respectively [4]. Taiwan has one of the highest hip fractures with a world population-adjusted incidence of 299/100,000 [5–7]. Between 2004 and 2011, a total of 141,397 Taiwanese aged 50 years and above had hip fractures. The incidence is estimated to increase 2.7-fold by 2035 [7]. Osteoporotic fractures are the major source of disability in many parts of the world and huge medical costs are incurred in their management. The high medical costs result from medical examinations, hospitalization, outpatient visits, treatments, and rehabilitation [8]. It is estimated that about 10–15 billion US dollars are spent annually in the management of osteoporotic fractures [8]. In Taiwan, the annual medication cost due to fractures increased 7.2-fold (from 8.1 to 58.9 million US dollars) between 1999 and 2010 [9].

Risk factors of osteoporosis are both modifiable (can be changed) and non-modifiable (cannot be changed). Some non-modifiable factors are age, genetics, sex, and menopausal status while some modifiable factors are lifestyle, BMI, and diet [10–13]. Some lifestyle and dietary factors associated with osteoporosis include coffee drinking, cigarette smoking, alcohol consumption, and exercise [10–13]. Coffee, one of the non-alcoholic beverages is substantially consumed worldwide [14, 15]. It contains many chemicals including caffeine, polyphenols as well as diterpenes [14, 15]. Previously, coffee was consumed mostly by the rich and celebrities in Taiwan. Nevertheless, its consumption has experienced a rapid increase recently as many people now incorporate it into their daily habits. For instance, in Taiwan, the import of coffee beans increased from about 4238 tons in 1998 to about 13,872 tons in 2007 [16]. Moreover, coffee-related products worth 28.2 billion NT dollars were imported in 2008 [16].

Results from previous studies on the association between coffee consumption and BMD or the risk of osteoporosis have not been consistent [17–20]. For instance, there was no significant association between coffee drinking and BMD among 200 postmenopausal Turkish women aged 40 years and above [17]. Similar results were observed in a study comprising 1761 premenopausal Korean women with a mean age of 36 years [18]. However, high coffee drinking was significantly associated with a small decrease in bone density but not an increased osteoporotic fracture risk among 5022 Swedish women aged over 40 years [19]. On the contrary, moderate coffee consumption was significantly associated with increased BMD among 4066 postmenopausal Korean women whose mean age was 62.6 years [20]. Because of these controversies, more investigations are warranted. Therefore, this longitudinal study was performed to assess the effect of coffee drinking on bone health of Taiwanese adults.

## Methods

### Data source and participants

Data used in the current longitudinal study were retrieved from the Li-Shin (Landseed) Hospital, Taoyuan, Taiwan. Individuals were included in the study if they lived in the Pinzheng District and were 30 years and above by December 31, 2005. In 2005, a total of 15,688 individuals were randomly sampled and invited for the Li-Shin prospective cohort study. Sampling was done using the proportionate stratified random sampling technique. Details about this sampling technique have been previously described [21]. Individuals were stratified by age and sex and the sample size of each group was proportionate to its size in the 2005 Pinzheng District population data. A total of 6152 individuals agreed to participate in this study.

**Fig. 1:**
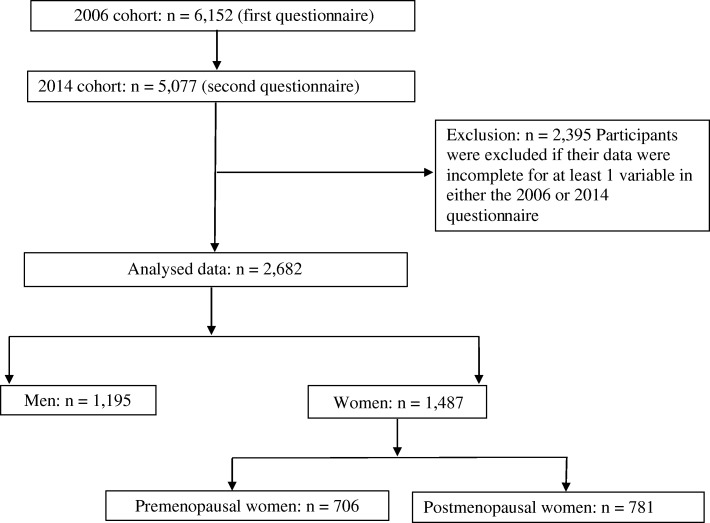
Study flow chart

In 2006, the 6152 individuals completed a questionnaire on lifestyle habits including coffee, alcohol drinking, exercise, diet, supplements intake, as well as smoking. They also provided information on their age, sex, educational level, and personal disease history (Additional file 1). Their bone health was determined as described below. In 2014, a total of 5077 individuals were followed up. Nonetheless, 2395 individuals were excluded from the study due to incomplete (missing) data. That is, any individual whose data were missing for at least 1 variable in either the 2006 or 2014 questionnaires were automatically excluded from the final analyses. Data from 2682 participants comprising 1195 men and 1487 women (706 premenopausal and 781 postmenopausal) were used in the final analyses (Fig. 1).

Bone health was assessed by quantitative ultrasound (QUS) using Acoustic Osteo Screener, AOS-100 (Aloka, Co. LTD, Tokyo, Japan). Details about the AOS-100 QUS device are described elsewhere [22–25]. In brief, the AOS-100 generates the index of bone density which is measured as the speed of sound (SOS) and the index of bone structure which is measured as the transmission index (TI) [24]. The osteo-sono assessment index (OSI) which reflects the bone stiffness in the calcaneus is derived from the SOS and TI as OSI = TI × SOS^2^ [22–25]. T-scores are generated by comparing the OSI with the mean OSI of a reference population [24, 25]. The OSI has been shown to be strongly correlated with BMD measured by dual X-ray absorptiometry (DXA) [23]. Moreover, it is highly reproducible and has a short-term coefficient of variation of < 3% [2, 26, 27].

The weekly frequency of coffee consumption in 2006 (start of the study) and 2014 (follow-up) was determined by asking participants how many times (≥7, 5–6, 3–4, 1–2 and 0) they drank coffee per week. A five-point scale (1–5) was used to represent this weekly frequency. That is, 1, 2, 3, 4, and 5 represented ≥7, 5–6, 3–4, 1–2 and 0 time(s)/week, respectively. One-time coffee drinking was equivalent to one cup (approximately 240 mL). Coffee consumption was categorised as “no, medium, and high” by comparing the five-point scale in 2006 with that in 2014. The comparison, 5/5 (i.e. 5 in 2006 and 5 in 2014) was defined as “no” consumption. Moreover, 1/4, 1/5, 2/4, 2/5, 3/4, 3/5, 4/4, 4/5 or 5/4 was defined as “medium” consumption while 1/1, 1/2, 1/3, 2/1, 2/2, 2/3, 3/1, 3/2, 3/3, 4/1, 4/2, 4/3, 5/1, 5/2 or 5/3 was defined as “high” consumption. The comparison, 5/5 for example, means the participant did not drink coffee in both 2006 and 2014 while 1/2 means a participant drank coffee ≥7 times per week in 2006 and 5–6 times per week in 2014.

Informed consents were obtained from all participants and all methods were carried out in accordance with relevant guidelines and regulations. Ethical approval for this study was issued by the institutional review board of Li-Shin Hospital (LSHIRB No./Protocol No.16–029-B1).

### Statistical analyses

The basic characteristics of the study participants were compared using the Chi-square test. The association between coffee consumption and T-scores was determined using multiple linear regression analysis. Results were presented as regression coefficients (β) and were considered statistically significant if the *P*-value was strictly less than 0.05 (*P* < 0.05). Adjustments were made for confounders including age, BMI, smoking, and drinking, among others. The analyses were performed using SAS 9.4 (SAS Institute, Cary, NC, USA).

## Results

Tables 1 and 2 show the basic characteristics of participants stratified by sex and menopausal status, respectively. There were significant differences between the number of male and female participants based on coffee drinking, T-scores, age, educational level, BMI, smoking, drinking, vegetarian diet, vitamin intake, and calcium intake (Table 1). Moreover, there were significant differences between the number of premenopausal and postmenopausal women based on coffee drinking, T-scores, age, educational level, BMI, exercise, diabetes, hypertension, heart disease, hyperlipidemia, and calcium intake (Table 2).

**Table 1:** Basic characteristics of both male and female participants

Variables	Men (*n* = 1195)	Women (*n* = 1487)	*P*-value
Coffee drinking			0.0382*
No	681(56.99)	822(55.28)	
Medium	265(22.18)	296(19.91)	
High	249(20.84)	369(24.82)	
T-score			0.0004*
*T* ≥ − 1	477(39.92)	679(45.66)	
-2.5 < *T* < −1	429(35.90)	430(28.92)	
*T* ≤ −2.5	289(24.18)	378(25.42)	
Age group (years)			<.0001*
30–49	471(39.41)	681(45.80)	
50–69	516(43.18)	754(50.71)	
≥ 70	208(17.41)	52(3.50)	
Education			<.0001*
College and above	367(30.71)	243(16.34)	
Senior high school	403(33.72)	475(31.94)	
Junior high school	182(15.23)	278(18.70)	
Elementary and below	243(20.33)	491(33.02)	
BMI (kg/m^2^)			<.0001*
Underweight	25(2.09)	51(3.43)	
Normal	462(38.66)	756(50.84)	
Overweight	423(35.40)	375(25.22)	
Obese	285(23.85)	305(20.51)	
Smoking			<.0001*
Never	597(49.96)	1438(96.70)	
Quit	174(14.56)	15(1.01)	
Yes	424(35.48)	34(2.29)	
Drinking			<.0001*
Never	852(71.30)	1430(96.17)	
Quit	53(4.44)	5(0.34)	
Yes	290(24.27)	52(3.50)	
Exercise			0.6398
No	421(35.23)	511(34.36)	
Yes	774(64.77)	976(65.64)	
Vegetarian diet			0.0025*
No	1107(92.64)	1327(89.24)	
Yes	88(7.36)	160(10.76)	
Personal disease history			
Diabetes	88(7.36)	76(5.11)	0.0155*
Hypertension	231(19.33)	211(14.19)	0.0004*
Heart disease	64(5.36)	48(3.23)	0.0062*
Hyperlipidemia	84(7.03)	80(5.38)	0.0764
Stroke	16(1.34)	2(0.13)	0.0001*
Supplement intake			
Vitamin D			0.6202
No	1168(97.74)	1449(97.44)	
Yes	27(2.26)	38(2.56)	
Other vitamins			<.0001*
No	1100(92.05)	1293(86.95)	
Yes	95(7.95)	194(13.05)	
Calcium			<.0001*
No	1002(83.85)	1016(68.33)	
Yes	193(16.15)	471(31.67)	

*P< 0.05

**Table 2:** Basic characteristics of premenopausal and postmenopausal women

Variables	Premenopausal women *n*(%)	Postmenopausal women *n*(%)	*P*-value
Coffee drinking			<.0001*
No	332 (47.03)	490 (62.74)	
Medium	146 (20.68)	150 (19.21)	
High	228 (32.29)	141 (18.05)	
T-score			<.0001*
T ≥ −1	410 (58.07)	269 (34.44)	
-2.5 < T < −1	115 (16.29)	315 (40.33)	
T ≤ −2.5	181 (25.64)	197 (25.22)	
Age group (years)			<.0001*
30–49	607 (85.98)	74 (9.48)	
50–69	99 (14.02)	655 (83.87)	
≥ 70	0 (0.00)	52 (6.66)	
Education			<.0001*
College and above	197 (27.90)	46 (5.89)	
Senior high school	322 (45.61)	153 (19.59)	
Junior high school	127 (17.99)	151 (19.33)	
Elementary and below	60 (8.50)	431 (55.19)	
BMI (kg/m^2^)			<.0001*
Underweight	35 (4.96)	16 (2.05)	
Normal	419 (59.35)	337 (43.15)	
Overweight	145 (20.54)	230 (29.45)	
Obese	107 (15.16)	198 (25.35)	
Smoking			0.1044
Never	677 (95.89)	761 (97.44)	
Quit	11 (1.56)	4 (0.51)	
Yes	18 (2.55)	16 (2.05)	
Drinking			0.0532
Never	670 (94.90)	760 (97.31)	
Quit	3 (0.42)	2 (0.26)	
Yes	33 (4.67)	19 (2.43)	
Exercise			0.0001*
No	278 (39.38)	233 (29.83)	
Yes	428 (60.62)	548 (70.17)	
Vegetarian diet			0.0661
No	641 (90.79)	686 (87.84)	
Yes	65 (9.21)	95 (12.16)	
Personal disease history			
Diabetes	13 (1.84)	63 (8.07)	<.0001*
Hypertension	45 (6.37)	166 (21.25)	<.0001*
Heart disease	15 (2.12)	33 (4.23)	0.0221*
Hyperlipidemia	18 (2.55)	62 (7.94)	<.0001*
Stroke	1 (0.14)	1 (0.13)	0.9430
Supplement intake			
Vitamin D			0.9891
No	688 (97.45)	761 (97.44)	
Yes	18 (2.55)	20 (2.56)	
Other vitamins			0.0619
No	626 (88.67)	667 (85.40)	
Yes	80 (11.33)	114 (14.60)	
Calcium			<.0001*
No	534 (75.64)	482 (61.72)	
Yes	172 (24.36)	299 (38.28)	

*P< 0.05

Tables 3, 4 and 5 show the association between coffee drinking and T-scores after adjustments were made for multiple confounders including age, BMI, smoking, and drinking, among others. Among all participants, medium and high coffee drinking were associated with higher T-scores, hence lower risk of osteoporosis. The regression coefficients (β) were 0.049; *P* = 0.3523 and 0.158; *P* = 0.0038 for medium and high coffee drinking, respectively (Table 3). That is, compared to non-drinkers, T-scores among medium and high coffee drinkers were higher and the differences were 0.049 and 0.158, respectively. Even though the results among medium drinkers were not statistically significant, a significant trend (*P* = 0.0046) was observed. That is, T-scores got higher as coffee consumption increased. In addition to coffee drinking, overweight, obesity, and exercise were significantly associated with higher T-scores (β = 0.181; *P* = 0.0021, β = 0.223; *P* = 0.0049, and β = 0.096; *P* = 0.0305, respectively). However, increased age, male sex, lower educational level (senior high school and below), and underweight were significantly associated with lower T-scores, hence higher osteoporosis risk. The regression coefficients (β) were − 0.447; *P* < .0001 for age 50–59 years, − 0.619; *P* < .0001 for age ≥ 70 years, − 0.200; *P* = 0.0008 for male sex, − 0.140; *P* = 0.0131 for senior high school, − 0.257; *P* = 0.0001 for junior high school, − 0.312; *P* < .0001 for elementary school and below, and − 0.314; *P* = 0.0141 for underweight.

**Table 3:** Multiple linear regression analysis showing the association between coffee drinking and T-scores among study participants

Variables	β	*P*-value
Coffee drinking (Reference: No)	–	
Medium	0.049	0.3523
High	0.158	0.0038*
P-trend	0.0046*	
Age group (Reference: 30–49 years)		
50–69	− 0.447	<.0001*
≥ 70	− 0.619	<.0001*
Sex (Reference: Women)		
Men	−0.200	0.0008*
Education (Reference:=College and above)		
Senior high school	−0.140	0.0131*
Junior high school	−0.257	0.0001*
Elementary and below	−0.312	<.0001*
BMI (Reference: Normal)		
Underweight	−0.314	0.0141*
Overweight	0.181	0.0021*
Obese	0.223	0.0049*
Smoking (Reference: Never)		
Quit	−0.017	0.8427
Yes	−0.094	0.1546
Drinking (Reference: Never)		
Quit	−0.038	0.7940
Yes	0.012	0.8607
Exercise (Reference: No)		
Yes	0.096	0.0305*
Vegetarian diet (Reference: No)		
Yes	−0.001	0.9729
Personal disease history		
Diabetes	−0.185	0.0817
Hypertension	0.037	0.5663
Heart disease	0.038	0.7150
Hyperlipidemia	−0.016	0.8530
Stroke	0.349	0.1682
Supplement intake		
Vitamin D (Reference: No)		
Yes	0.006	0.9631
Other vitamins (Reference: No)		
Yes	0.104	0.2160
Calcium (Reference: No)		
Yes	−0.105	0.0852

*P< 0.05. Adjusted for age, education, BMI, smoking, drinking, exercise, vegetarian diet, vitamin D, other vitamins, calcium, blood type, fasting blood glucose, creatinine, uric acid, total cholesterol, triglycerides, HDL, LDL, GOT, GPT, SBP, DBP, waist circumference, waist-hip ratio, body fat, diabetes, hypertension, heart disease, hyperlipidemia and stroke

**Table 4:** Multiple linear regression analysis showing the association between coffee drinking and T-scores among men and women

Variables	Men		Women	
	β	*P*-value	β	*P*-value
Coffee drinking (Reference: No)				
Medium	0.003	0.9662	0.105	0.1445
High	0.237	0.0067*	0.101	0.1492
P-trend	0.0161*		0.0947	
Age group (Reference: 30–49 years)				
50–69	−0.334	<.0001*	−0.388	<.0001*
≥ 70	−0.482	<.0001*	−0.829	<.0001*
Education (Reference: College and above)				
Senior high school	−0.085	0.2740	−0.169	0.0458
Junior high school	−0.245	0.0135*	−0.245	0.0116*
Elementary and below	−0.228	0.0246*	− 0.248	0.0124*
BMI (Reference: Normal)				
Underweight	−0.369	0.0775	− 0.258	0.1133
Overweight	0.272	0.0011*	0.082	0.3383
Obese	0.325	0.0049	0.111	0.3172
Smoking (Reference: Never)				
Quit	−0.086	0.3410	0.340	0.3238
Yes	−0.128	0.0800	0.211	0.2604
Drinking (Reference: Never)				
Quit	−0.020	0.8973	0.190	0.7787
Yes	0.011	0.8865	−0.011	0.9495
Exercise (Reference: No)				
Yes	0.162	0.0172*	0.037	0.5339
Vegetarian diet (Reference: No)				
Yes	−0.059	0.4502	0.025	0.4599
Personal disease history				
Diabetes	−0.236	0.1169	−0.129	0.4031
Hypertension	0.004	0.9657	0.049	0.6030
Heart disease	0.118	0.4049	−0.065	0.6822
Hyperlipidemia	−0.013	0.9183	0.084	0.5012
Stroke	0.029	0.9162	1.564	0.0177*
Supplement intake				
Vitamin D (Reference: No)				
Yes	0.053	0.8028	−0.049	0.7669
Other vitamins (Reference: No)				
Yes	0.377	0.0167*	−0.022	0.8250
Calcium (Reference: No)				
Yes	−0.272	0.0159*	−0.053	0.4692

*P< 0.05. Adjusted for age, education, BMI, smoking, drinking, exercise, vegetarian diet, vitamin D, other vitamins, calcium, blood type, fasting blood glucose, creatinine, uric acid, total cholesterol, triglycerides, HDL, LDL, GOT, GPT, SBP, DBP, waist circumference, waist-hip ratio, body fat, diabetes, hypertension, heart disease, hyperlipidemia and stroke

**Table 5:** Multiple linear regression analysis showing the association between coffee drinking and T-scores among premenopausal and postmenopausal women

Variables	Premenopausal women		Postmenopausal women	
	β	*P*-value	β	*P*-value
Coffee drinking (Ref: No)				
Medium	0.233	0.0355*	0.037	0.7061
High	0.234	0.0152*	−0.088	0.4108
P-trend	0.0108*		0.5399	
Age group (Ref: 30–49 years)				
50–69	−0.218	0.0881	−0.565	<.0001*
≥ 70	–	–	−0.983	<.0001*
Educational (Ref: College and above)				
Senior high school	−0.249	0.0123*	−0.010	0.9563
Junior high school	−0.255	0.0510	−0.195	0.2658
Elementary and below	−0.388	0.0255*	−0.123	0.4573
BMI (Ref: Normal)				
Underweight	−0.134	0.5282	−0.430	0.1036
Overweight	0.209	0.1216	0.033	0.7723
Obese	0.218	0.2334	0.052	0.7139
Smoking (Ref: Never)				
Quit	0.668	0.0808	−0.801	0.4042
Yes	0.465	0.0605	−0.148	0.6212
Drinking (Ref: Never)				
Quit	0.096	0.8942	–	–
Yes	−0.025	0.9083	−0.048	0.8694
Exercise (Ref: No)				
Yes	−0.028	0.7542	0.090	0.2737
Vegetarian diet (Ref: No)				
Yes	0.024	0.7706	0.019	0.6070
Personal disease history				
Diabetes	−0.306	0.3908	− 0.244	0.1732
Hypertension	0.327	0.1440	−0.052	0.6207
Heart disease	0.241	0.3829	−0.187	0.3447
Hyperlipidemia	−0.295	0.2992	0.206	0.1420
Stroke	0.200	0.8335	2.624	0.0055*
Supplements				
Vitamin D (Ref: No)				
Yes	−0.242	0.3356	0.045	0.8370
Other vitamins (Ref: No)				
Yes	−0.116	0.5060	0.046	0.7087
Calcium (Ref: No)				
Yes	0.039	0.7670	−0.088	0.3244

*P < 0.05. Adjusted for age, education, BMI, smoking, drinking, exercise, vegetarian diet, vitamin D, other vitamins, calcium, blood type, fasting blood glucose, creatinine, uric acid, total cholesterol, triglycerides, HDL, LDL, GOT, GPT, SBP, DBP, waist circumference, waist-hip ratio, body fat, diabetes, hypertension, heart disease, hyperlipidemia and stroke

When stratified by sex, coffee drinking was associated with higher T-scores in both men and women (Table 4). However, significant results were prominent only among men with medium coffee drinking (β = 0.237; *P* = 0.0067). A significant trend (*P* = 0.0161) was also prominent only among men. Overweight, exercise, and vitamin intake were also significantly associated with higher T-scores among men (β = 0.272; *P* = 0.0011, β = 0.162; *P* = 0.0172, and β = 0.377; *P* = 0.0167, respectively). Nevertheless, increased age and lower educational level (junior high school and below) were significantly associated with lower T-scores among both sexes. Among men, the regression coefficients (β) were − 0.334; *P* < .0001 for age 50–60 years, − 0.482; *P* < .0001 for age ≥ 70 years, − 0.245; *P* = 0.0135 for junior high school and − 0.228; *P* = 0.0246 for elementary school and below. Among women, regression coefficients (β) were − 0.388; *P* < .0001 for age 50–60 years, − 0.829; *P* < .0001 for age ≥ 70 years, − 0.245; *P* = 0.0116 for junior high school and − 0.248; *P* = 0.0124 for elementary school and below.

Based on menopausal status, both medium and high coffee drinking were significantly associated with higher T-scores among premenopausal women (β = 0.233; *P* = 0.0355 for medium and β = 0.234; *P* = 0.0152 for high coffee drinking). In addition, a significant trend (*P* = 0.0108) was prominent (Table 5). Increased age was significantly associated with lower T-scores in postmenopausal women (β = − 0.565; *P* < .0001 and β = − 0.983; *P* < .0001 for age 50–69 and ≥ 70 years, respectively). However, lower educational level (senior high school and elementary school and below) was significantly associated with lower T-scores only among premenopausal women (β = − 0.249; *P* = 0.0123 for senior high school and β = − 0.388; *P* = 0.0255 for elementary school and below).

## Discussion

In the current study, coffee drinking was significantly associated with higher T-scores among men and premenopausal women. As far as we know, this study is among the first to demonstrate the protective effect of coffee drinking on osteoporosis risk among premenopausal women. Although osteoporosis is more common in postmenopausal women, its occurrence in men and premenopausal women cannot be ignored. Therefore increasing the bone mass of men and both premenopausal and menopausal women can serve as a preventive measure against bone loss and subsequently osteoporosis [3]. The strength of the current study is that participants were stratified by sex and menopausal status using information obtained from two questionnaires (2006 and 2014) and analysis was done using the multiple linear regression model which adjusted for many confounders.

So far, the association between coffee drinking and BMD or the risk of osteoporosis has been incoherent. In line with the current study, moderate coffee consumption was significantly associated with increased BMD among 992 Chinese men with a mean age of 64.11 years [3]. Moreover, coffee consumption was associated with increased broadband ultrasound attenuation (BUA), hence a lower risk of osteoporosis among 344 Malaysian women aged 50 years and above [28]. Besides, coffee drinking at the premenopausal stage was not significantly associated with BMD among 200 postmenopausal Turkish women aged 40 years [17]. Furthermore, in a cross-sectional study involving 1336 premenopausal and 1593 postmenopausal Taiwanese aged 30 years and above, coffee consumption was significantly associated with decreased risk of osteoporosis in premenopausal, but not postmenopausal women [29]. Unlike the current study, coffee drinking was not significantly associated BMD among 1761 premenopausal Korean women with a mean age of 36 years [18]. Moreover, it was significantly associated with decreased BMD among 258 healthy Polish men aged 40–63 years [30]. In addition, it was significantly associated with decreased BMD among men in a Swedish cohort comprising 359 men and 358 women aged 70 years [31]. It was also associated with lower BMD among 100 postmenopausal women aged 50–65 years who lived in Sarajevo [32]. Furthermore, high coffee drinking was significantly associated with a small decrease in bone mineral density but not an increased osteoporotic fracture risk among 5022 Swedish women aged over 40 years [19]. Moderate coffee consumption was significantly associated with increased BMD among 4066 postmenopausal Korean women with a mean age of 62.6 years [20]. Moreover, coffee consumption was significantly associated with increased BMD among 1817 Chinese postmenopausal women [33].

The mechanism behind the beneficial effects of coffee on bone health is still unclear. However, it can be partly explained in terms of its antioxidant and anti-inflammatory properties. For instance, coffee is composed of high polyphenols (chlorogenic acids) which have the potential to inhibit osteoclastogenesis hence, reduction of osteoporosis risk [3, 20, 34].

The use of QUS might be one of the factors contributing to the discrepancies between this study and previous ones. Moreover, these discrepancies can be accounted for in terms of differences in sample sizes, ethnicities, study designs, statistical methods, ages, sex, as well as confounders. For instance, in this study, coffee drinking was associated with higher T-scores. The study was longitudinal in nature and participants included both Taiwanese men (*n* = 1195) and women (*n* = 1487) aged 30 years and above. The female participants were further stratified by menopausal status. Moreover, linear regression was used to determine the association between T-scores and coffee drinking and adjustments were made for important confounders like age, BMI, smoking, exercise, and diet, among others. However, in a study conducted by Choi and colleagues, there was no significant association between coffee drinking and BMD. The study was cross-sectional in nature and participants comprised only Korean premenopausal women (*n* = 1761) with a mean age of 36 [18]. In another previous study, coffee consumption was significantly associated with decreased BMD. Participants consisted of 258 Polish men aged 40–63 years. The relationship between coffee and BMD was determined by analysis of variance (ANOVA) and no adjustments were made for confounders [30]. Furthermore, in another study where both men (*n* = 359) and women (*n* = 358) aged 70 years were included, coffee drinking was significantly associated with decreased BMD. Moreover, the effect of cytochrome P450 1A2 (CYP1A2) genotype was considered in the study [31].

The frequency of coffee consumption is important when determining the relationship between coffee consumption and bone health. For instance, compared to 1 cup per day, the consumption of 4 or more cups of coffee per day was found to be significantly associated with increased osteoporotic fracture risk among 31,527 Swedish women aged 40–76 years with low calcium consumption [35]. Moreover, the consumption of 4 or more cups of coffee per day led to a reduction in male femoral BMD compared with lower volumes. However, there was no such observation in women [31]. In a cohort of Swedish women aged above 40 years, consuming 4 or more cups of coffee per day led to decreased bone density. However, this did not increase the osteoporotic fracture risk [19]. Similarly, in a cohort of Swedish men, consuming 4 or more cups of coffee per day was not associated with osteoporotic fracture risk [36]. In the current study, the frequency of coffee consumption was also considered. The questionnaires used contained information on how many times (≥7, 5–6, 3–4, 1–2 and 0) participants consumed coffee in a week and a five-point scale was used to represent this weekly frequency as stated in the methods section. The grouping of coffee drinking into “no, medium and high” was based on a combination of the weekly frequency of consumption in both 2006 and 2014 questionnaires.

In line with this study, exercise  has been previous demonstrated to be associated with higher BMD [37–40]. It would be better to have assessed the relation between weight-bearing exercise and calcaneal OSI in detail. However, this study did not focus on the exercise type. Further investigation taking the exercise type into consideration is recommended. Increased BMD was also associated with obesity and overweight [28, 40, 41]. The association of obesity/overweight with BMD is probably due to the conversion of androgen to estrogen which increases bone mass [40, 42]. Another plausible explanation is the higher mechanical loading in obese and overweight individuals (higher BMI) [28, 42]. Low BMI [41, 43, 44], age [3, 6, 39, 43, 44] and education below college level [39, 44, 45] have been associated with lower BMD. Low bone mass is associated with increased age because as time progresses, the bone tissue’s synthesizing capacity reduces [44].

The limitation of this study is that bone health was assessed by QUS. Unlike DXA, bone health assessment using QUS devices have a lot of variations as far as precision, T-score, reference ranges are concerned [2, 46]. Therefore, in clinical practice, QUS is not a gold standard for BMD measurement [2, 46]. It only serves as a screening guide for patients to go for a more definitive test like DXA. However, OSI measured by QUS has been shown to be closely correlated (*r* = 0.87) with BMD measured by DXA [23]. Furthermore, QUS devices are non-ionizing (safe), portable and cost-effective. Nonetheless, the use of QUS devices in assessing the risk of fracture is very pertinent in developing countries especially in the absence of DXA.

In conclusion, coffee drinking was significantly associated with higher T-scores, hence a lower risk of osteoporosis in men and premenopausal women. Future studies using DXA are recommended to confirm these findings.

## Additional file

Additional file 1: Translated questionnaire. (PDF 1664 kb)

## Abbreviations

ANOVA: Analysis of variance analysis
AOS: Acoustic Osteo-screener
BMD: Bone mineral density
BMI: Body mass index
BUA: Broadband ultrasound attenuation
CYP1A2: Cytochrome P450 1A2
DBP: Diastolic blood pressure
DXA: Dual-energy X-ray absorptiometry
GOT: Glutamic-oxaloacetic transaminase
GPT: Glutamic-pyruvic transaminase
HDL: High-density lipoprotein
LDL: Low-density lipoprotein
QUS: Quantitative ultrasound
SBP: Systolic blood pressure
