# Supplementary material for: Does coffee drinking have beneficial effects on bone health of Taiwanese adults? A longitudinal study
Source: BMC Public Health. 2018 Nov 20;18:1273. doi: 10.1186/s12889-018-6168-0 (PMC6245613; doi:10.1186/s12889-018-6168-0)
Supplement: Supplementary file 1 — Translated questionnaire. (PDF 1664 kb) [file 12889_2018_6168_MOESM1_ESM.pdf]

壢新醫院社區複合式健康篩檢服務問卷

(Landseed Hospital Community Health Screening Service Questionnaire)

注意事項(Instructions)

※ **受檢民眾(Participant)**

1. **請確認您的問卷封面上欄位資料是否正確(如有錯誤請您通知工作人員更正)**

   (Please confirm that the filled information on the cover of your questionnaire is correct, please notify the staff to correct if there is any error)

2. **填答方式: 請選擇您所最合適答案並在該欄位上「○」欄位內塗滿**

   (How to answer: Please select the answer that is most suitable for you and fill it in the "○" field.)

※ **服務人員(Interviewer):**

1. **發放問卷時請確實核對民眾檢查單(黃單)基本資料(姓名、篩編)**

   (Please check the basic information (name, screening number) of the examination list (in yellow)

2. **會員身分別確認無誤後發放問卷乙份**

   (Confirm the identity before giving the questionnaire)

3. **取下條碼 1 張黏貼於問卷封面受檢者篩檢編號欄位(如無條碼可以手寫方式寫入篩編)**

   (Remove 1 barcode and paste it on the cover of the questionnaire.)

4. **填寫受檢者姓名。**

   (Fill in the name of the participant)

5. **請確認填答者，並在填答欄位上正確勾選(空格也請填入)。**

   (Please confirm the participant and check it correctly in the answer field (please fill in the space as well).)

受檢者姓名(Participant's name)：___________________

受檢者篩檢編號(Participant's screening number)：____

填答者(Respondent)：

1 自行填答 (Answer yourself)

2 訪員(Interviewer) 姓名(Name)：__________________________

3 家屬代答 (Participant’s relative) 與受檢者的關係(Relationship)：________

問卷填寫開始時間：______時______分

Start of Interview: _____Hour____Minute

第一部份：生活型態(Part 1: Lifestyle)

1 您有無規律吸菸的習慣？ (Do you have a habit of smoking regularly?)

0 沒有(No)

1 現在沒有但過去有吸(Quit)【**續問**(Continued)】,

______歲開始規律吸菸(How old were you when you started smoking regularly?),

平均一天吸____支(How many cigarettes a day did you smoke?),

戒菸____年(How long have you quit smoking?)

2 目前還有吸(Yes)【**續問**(Continued)】

______歲開始規律吸菸(How old were you when you started smoking regularly?),

平均一天吸_____支(How many cigarettes a day do you smoke?)

2.1 請問您是否覺得吸入過多二手菸？(Do you feel that you inhale too much secondhand smoke?) 1 無 (No)【**跳答第 3 題**】**(Skip to question 3)** **3** 偶爾 (Sometimes) 5 經常 (Usually)

2.2 二手菸的主要來源？(What is the main source of the secondhand smoke?) 1 工作 (Workplace) 2 家中 (Home) 3 公共場所 (Public place)

3 您有無規律喝酒習慣？ (Do you have a habit of drinking alcohol regularly?)

0 無(No)(包含平時不喝但應酬才喝) (Including drinking when you are socializing)

1 現在沒有但過去有(Quit) 【**續問**(Continued)】

_____歲開始喝(How old were you when you started to drink?),

戒酒_____年(How long have you quit drinking?)【**續問**(Continued)】

2 有固定飲酒習慣(Yes) 【**續問**(Continued)】

1 每天小酌 (Drink daily) (約一罐 350c.c 台啤/1 天 (A can of beer is about 350c.c/day))

2 每天大量飲酒 (Drink a lot every day) ， 請勾選您最常飲酒種類及份量 (Please choose the type and quantity of alcohol you drink most.)

1 啤酒(beer)、保力達 B(“Paolyta-B”)

每天約____杯(約 250c.c/杯) ___cups per day (about 250 c.c / cup)

2 玫瑰紅、葡萄酒 (red wine), 紹興 (“Shaoxing” wine), 米酒 (rice wine)

每天約____杯(約 250c.c/杯) ___cups per day (about 250 c.c / cup)

3 **烈酒類** spirits (**高粱**) (“Gaoliang” wine)、**洋酒**、**伏特加**(vodka)、**茅台**)

每天約____杯(約 10c.c/杯) ___cups per day (about 10 c.c / cup)

_____歲開始喝(How old were you when you started to drink?)【**續問**(Continued)】

4 您有無規律嚼檳榔習慣？(Do you have a habit of chewing betel nut regularly?) 0 沒有 (No)

1 現在沒有但過去有嚼 (Quit)【**續問**】(Continued)_歲開始嚼 (How old were you when you started to chew) 平均一天嚼 顆 (what is the average number of betel nuts you chewed per day), 戒_____年 (How long have you quit chewing)

2 目前有嚼 (Yes)【**續問**】**(Continued)**______歲開始嚼 (How old were you when you started to chew), 平均一天嚼_____顆 (what is the average number of betel nuts you chew per day)

5 請問您日常生活中刷牙的頻率？(How often do you brush your teeth per day?) 0 無 (No) 1 沒有每天 (Not everyday) 2 每天 1~2 次 (1-2 times) 3 每天三次以上 (3 times or more)

6 請問您是否有每月量體重的習慣？(Do you have a habit checking your weight monthly) 0 沒有 (No) 1 有 (Yes)

7 請問您從事何種工作(職業)？(What is your occupation?)__ （例如 (E.g)：噴殺蟲劑 (Spraying insecticides)、廚師 (Chef)、木工 (Carpenter)、服務生(Waiter)）

8 請問您目前從事職業活動量為？(How intensed is your current job) 1 輕度 (Mild) 【如 (E.g.) 家管 (Housewife)、開計程車 (Taxi driver)、售貨員(Salesperson), 焊接工 (Welder)、零件組合 (Mechanic) 】

2 中度 (Medium)【如 (E.g.)木匠 (Carpenter)、氣動工具操作 (Pneumatic tool operator) 】

3 重度 (Severe) 【如 (E.g.) 採礦劏煤 (Coal mining)、熔爐操作 (Furnace operation) 】

4 極重度 (Extremely severe)【如 (E.g.) 扛木柴 (Wood mover) 、水泥工 (Cement mover)、搬貨運 (Freight mover)】

9 運動習慣 (Exercise habit)

9.1 您目前休閒時有無規律運動習慣？ (Do you have regular exercise habits when you are at leisure?)

0 沒有(No)【請跳答第10題 (skip to question 10)】 1 有(Yes)【續問(Continued)】

9.2 平均一週運動_____次 (How many times do you exercise a week?) ， 每次____分鐘 (____minutes/per exercise)

9.3 您經常做的運動為何？ (What sports do you often do?) (可複選 Multiple answers are accepted.)

1 輕度運動(Light)：如園藝(Gardening), 高爾夫球 (Golf)、棒球(Baseball)、柔軟體操 (Soft Gymnastics) 、一般舞蹈 (General dance), 腳踏車(慢速) (Bicycle (slow speed)),

若非以上所列 (Others), 請填答 (Specify)：______________________________

2 中度運動(Moderate)：如籃球(Basketball)、排球(Volleyball), 乒乓球 (Table tennis), 羽毛球 (Badminton)、舞蹈(劇烈) Dance (Violent)、游泳(隨意) Swimming (casual)、快速走路 (Walking fast) ， 若非以上所列 (Others), 請填答 (Specify)：______________________________

3 重度運動(Heavy)：慢跑(8 公里/小時) Jogging (8 km / h), 爬山 (Climbing)、爬樓梯 (Climbing stairs)、游泳(自由式或仰式) Swimming (Freestyle or upright)) ，

若非以上所列 (Others), 請填答 (Specify)：______________________________

4 劇烈運動(Strenuous)：如跑步(12 公里/小時) Running (12 km / h), 跳繩 (Skipping rope)、賽舟 (Racing boat)、游泳(蝶式) Swimming (butterfly)、溜冰比賽 (Ice skating competition) ，

若非以上所列, 請填答(Others)：______________________________

10. 請問您是否經常煮飯？(Do you often cook?) 0 否 (No)【請跳答第 11 題 (Skip to 11)】 1 有 (Yes)，平均每星期____次 (What is the average number of times you cook per week) 【續問】 **(Continued)**

10.1 在煮飯時您覺得會吸入到過多油煙嗎？(Do you think you inhale too much fumes when cooking?)

0 不會 (No) 1 還好 (Somehow) (2 很多 (Too much)

11 請問您是否有規律燒香拜拜的習慣？(Do you have a habit of burning incense) 0 沒有 (No) 1 有 (Yes) 【續問】(Continued)

廟裡拜拜 (Do you worship in the temple?), 平均每月 ____ (How many times per month)

家中拜拜, Do you worship at home?), 平均每月 ____ (How many times per month)【續問】(Continued)

家中燒香拜拜的地方是不是通風良好？(Is the place of worship well ventilated) 0 不是 (No) 1 是 (Yes)

12 平常使用之交通工具？(What kind of transportation do you use?) (複選題) (You can choose more than one answer)

1 走路 (Walk)

2 腳踏車 (Bicycle)

3 機車 (Scooter)【續問】 (Continued) 1 很少戴安全帽 (Seldom wears the helmet) 2 幾乎都有戴 (Usually wears the helmet)

4 汽車 (Car)【續問】(Continued) 1 很少繫安全帶 (Seldom wears the safety belt) 2 幾乎都有繫 (Usually wears the safety belt)

5 大眾運輸 (Public transportation) {如 (E.g.):公車 (Bus)、火車 (Train)、高鐵 (High speed train)..............等等 (etc)}

13 飲水問題 (Water Source)

13.1 請問您家中喝的水源是？(What is the source of water in your home?) 1 自來水 (Tapwater) 2 地下水(井水) (Groundwater) (3 其他 (Others)________

13.2 請問您家中是否有裝水塔？ (Is there a water tank in your home?) 0 沒有 (No) 1 有 (Yes)

13.3 請問您家中是否有裝設飲水過濾器 (Does your home have a drinking water filter?) (如 (E.g.) 逆滲透 (Reverse osmosis)...等 (etc) 0 沒有 (No) 1 有(Yes)

14 手機使用情形 (Cellphone use)

14.1 請問您是否有使用手機？(Do you use a cellphone) 0 無 (No) 1 有 (Yes)【續問】(Continued)

14.2 平均每日通話時間？(What is the average duration you talk on phone a day) 1 約 0~10 分

鐘 (0-10 minutes)　　3　約 11~20 分鐘 (11-20 minutes)　　4　約 21~60 分鐘 ((21-60 minutes) 5　約超過 1 小時 (More than an hour)

第二部份：飲食習慣(Part 2: Eating habit)

1.您是不是素食者？ (Are you a vegetarian?) 0 不是(No) 1 是(Yes)

2.您平均每天吃幾碗飯或麵？ (What is the average quantity of rice or noodles you eat per day?) 0 沒吃 (None) 1 1-2 碗 (1-2 bowls) 2 3 碗 (3 bowls) 3 4 碗 (4 bowls) 4 5 碗以上 (5 and above)

3.您平均每天吃幾碗蔬菜？(What is the average quantity of vegetables you eat per day?) 0 沒吃 (None) 1 1/2 碗 (1/2 bowl) 2 1 碗 (1 bowl) 3 2 碗 (2 bowls) 4 3 碗 (3 bowls) 5 4 碗 (4 bowls)

4.您平均每天吃肉類的數量？ (What is the average quantity of meat you eat per day?) (1 份為半碗肉或一塊排骨或半支雞腿 (A serving means half a bowl of meat, a piece of pork rib or half a chicken leg) 0 沒有吃 (None) 1 1 份 (1 serving) 2 2 份 (2 servings) 3 3 份 (3 servings) 4 4 份 (4 servings)

5.您平均每天吃肉的類型為？(What kind of meat do you often eat per day?) 0 大塊肉 (Larger piece) (例如, (E.g.)：雞排 (Chicken steak)、豬排 (Pork steak)、牛排 (Beef steak)

1 小塊肉 (Smaller piece) (例如 (E.g.)：焢肉類 (Stewed pork rice)

2 丁狀 (Diced)、絲狀(Sliced)、碎肉 (Minced)

6.一般而言，您是否會吃的很鹹？(Do you generally eat salty food?)

1 非常清淡 (Very lightly) 2 清淡 (Lightly) 3 普通 (Averagely) 4 有點鹹 (A bit salty)

(5 很鹹 (Very salty)

7.您每天用餐時，是否有沾醬油或是其他含鹽份佐料的習慣？(Do you have the habit of adding soy sauce or other salt-containing ingredients in your daily meals?) (一份相當於 2 小湯匙醬油、番茄醬、辣椒醬、黑醋、1 小湯匙的胡椒鹽 (A serving means 2 tablespoons of soy, tomato and chili sauce, balsamic vinegar, and 1 tablespoon of pepper salt) 0 沒吃 (None) 1 1 份 (1 serving) 2 2 份 (2 servings) 3 3 份 (3 servings) 4 4 份 (4 servings)

8.您家裡炒菜最常用什麼油？(What cooking oil do you often use at home?) 0 不知道 (I do not know) 1 知道 (I know)【續問】(Continued)

8.1(填答家中最常使用油品並依頻率順序填寫 1.2.3.4… (Choose the most frequently used oil in your home and fill in the frequency order 1.2.3.4…) 順序:_(Order)_1.沙拉油 (Salad oil) 2.豬油 (Lard) 3.橄欖油 (Olive oil) 4.葵花油 (Sunflower oil) 5.花生油(Peanut oil) 6.玉米油 (Corn oil) 7.清香油 (Sesame oil) 8.外食或其它用油 (Eat from outside or use other types of oil)，如 (Specify)：

____

9. 請就您最近半年內的飲食習慣，回答下列問題： (Please answer the following questions about your eating habits during the last six months.)

	1. 每週七次以上 7 times or more/week	2. 每週五－六次 5-6 times / week	3. 每週三－四次 3-4 times / week	4. 每週一－二次 1-2 times / week	5. 很少吃或不吃 Rare or never
9.1 您吃早餐的情形？(How often do you eat breakfast?)	1	2	3	4	5
9.2 您早餐外食的情形？(How often do you eat breakfast outside?)	1	2	3	4	5
9.3 您吃午餐的情形？(How often do you eat lunch?)	1	2	3	4	5
9.4 您午餐外食的情形？(How often do you lunch outside?)	1	2	3	4	5
9.5 您吃晚餐的情形？(How often do you eat dinner?)	1	2	3	4	5
9.6 您晚餐外食的情形？(How often do you eat dinner outside?)	1	2	3	4	5
9.7 你吃宵夜的情形？(How often do you eat snacks?)	1	2	3	4	5
9.8 您除正餐外，有無吃西點、麵包、蛋糕或點心的情形？(In addition to your meals, do you eat bread, cake or snack?)	1	2	3	4	5
9.9 您除正餐外，有無吃中式點心如雞排、鹹酥雞的情形？In addition to your meals, do you eat Chinese snacks like chicken steak and crispy chicken?)	1	2	3	4	5

	1. 每週七次以上 7 times or more/week	2. 每週五－六次 5-6 times / week	3. 每週三－四次 3-4 times / week	4. 每週一－二次 1-2 times / week	5. 很少吃或不吃 Rare or never
9.10 您每星期吃幾次魚？(How many times do you eat fish per week?)	1	2	3	4	5
9.11 您每星期吃幾次海鮮(如蝦、蟹、花枝…等)？(How many times do you eat sea food per week? E.g., Shrimp, Crab, Squid etc.)	1	2	3	4	5
9.12 您每星期吃幾次豆類食品(豆腐、豆干…)或蛋製品？(How many times do you eat bean products e.g., bean curd, dry bean curd or egg product per week?)	1	2	3	4	5
9.13 您每星期吃幾次水果？(How many times do you eat fruits per week?)	1	2	3	4	5
9.14 您每星期喝幾次牛奶(一杯約 240 cc)？(How many times do you drink milk per week?: 1 cup = 240 cc)	1	2	3	4	5
9.15 您每星期喝幾次豆漿(一杯約 240 cc)？How many times do you drink soymilk per week?: 1 cup = 240 cc)	1	2	3	4	5
9.16 您每星期喝幾次優酪乳(一杯約 240 cc)？How many times do you drink yogurt per week?: 1 cup = 240 cc)	1	2	3	4	5
9.17 **您每星期喝幾次飲料(如汽水、可樂、果汁、蘆筍汁)？**How many times do you drink beverages per week? (such as soda, cola, juice, asparagus juice)	1	2	3	4	5
9.18 您每星期喝幾次咖啡(一杯約 240 cc)？(How many times do you	1	2	3	4	5

	1. 每週七次以上 7 times or more/week	2. 每週五－六次 5-6 times / week	3. 每週三－四次 3-4 times / week	4. 每週一－二次 1-2 times / week	5. 很少吃或不吃 Rare or never
drink coffee a week? (a cup of about 240cc)<br>喝咖啡時的習慣(Habit of drinking coffee)<br>黑咖啡(black coffee) 0 不是 (No) 1 是 (Yes)<br>加糖(add sugar) 0 不是 (No) 1 是 (Yes)<br>加奶精(add creamer) 0 不是 (No) 1 是 (Yes)					
9.19 您每星期喝幾次茶(一杯約 240 cc) (How many times do you drink tea a week? (a cup of about 240cc)<br>茶的種類 (What kind of tea?)：________________(如 (E.g.) 紅茶 (Black tea)、綠茶 (Green tea, …)<br>喝茶時的習慣 (Habit of drinking tea) 無糖無奶精 (Sugar and creamer-free) 0 不是 (No) 1 是 (Yes)<br>加糖 (Add sugar) 0 不是 (No) 1 是 (Yes)	1	2	3	4	5

	1. 每週七次以上 7 times or more/week	2. 每週五－六次 5-6 times / week	3. 每週三－四次 3-4 times / week	4. 每週一－二次 1-2 times / week	5. 很少吃或不吃 Rare or never
加奶精 (Add creamer) 0 不是 (No) 1 是 (Yes)					
9.20 您每星期吃幾次泡麵的次數？(How many times do you eat instant noodles per week?)	1	2	3	4	5

第三部份：家族病史 (Part Three: Family Disease History)

請問您的**祖父母、外祖父母、父母、子女、兄弟姐妹**之中有沒有人得過下列疾病？請填入稱謂。

填寫範例 (Does any of your grandparents, parents, children, brothers and sisters have the following diseases? Please fill in the blanks as shown:)

祖 父 (Grandfather)				√												

一、癌症 (1: Cancer)

1 全無下述所列之癌症 (None) 0 有 (Yes) (請填寫下列表單) (Please fill the form below)

**親屬稱謂** (Relationship)	(1)	(2)	(3)	(4)	(5)	(6)	(7)	(8)	(9)	(10)	(11)	(12)	(13)	(14)	(15)	(16)
	子宮頸癌 (Cervical cancer)	乳癌 (Breast cancer)	肝癌 (Liver cancer)	肺癌 (Lung cancer)	大腸直腸癌 (Colorectal cancer)	胃癌 (Gastric cancer)	口腔癌 (Oral cancer)	攝護腺癌 (Prostate cancer)	胰臟癌 (Pancreatic cancer)	膽囊癌 (Gallbladder cancer)	卵巢癌 (Ovarian cancer)	鼻咽癌 (Nasopharyngeal carcinoma)	食道癌 (Esophageal cancer)	骨癌 (Bone cancer)	白血病（血癌）(Leukemia)	其他癌症（請註明）Others (Specify)

二、慢性疾病 (2 :Chronic Diseases)

1 全無下述所列之癌症 (None) 0 有 (Yes) (請填寫下列表單) (Please fill the form below)

親屬稱謂(Relationship)	(1) 糖尿病(Diabetes)	(2) 高血壓(Hypertension)	(3) 腦血管疾病/中風(Cerebrovascular disease/stroke)	(4) 心臟血管疾病(Cardiovascular disease)	(5) 腎臟病(含洗腎) (Kidney disease)	(6) 慢性肝炎/肝硬化 (Chronic hepatitis/cirrhosis)	(7) 攝護腺腫大(Prostate hypertrophy)	(8) 痛風(Gout)	(9) 巴金森氏症(Parkinson's disease)	(10) 癲癇(Epilepsy)	(11) 精神疾病(Mental illness)	(12) 結核病(Tuberculosis)	(13) B\C型肝炎帶原(Hepatitis B and C)	(14) 氣喘(Asthma)	(15) 家族性大腸瘜肉症 (Familial adenomatous poliposis)

第四部份：個人病史(Part 4: Personal Disease History)

有無疾病(Disease situation)				治療方式?(可複選) (What is your treament method) (Can check multiple choices)							是否規則治療? (Do you take your treatment regularly)				
請問您有沒有下列疾病？ (Do you have any of the following diseases?)	0. 無 (No)	1. 有 (Yes)	患病幾年 (Duration of sickness)	0. 無 (None)	1. 西醫 (Medical doctor)	2. 中醫 (Traditional Chinese medicine practitioner)	3. 西藥房 (Pharmacy)	4. 中藥 (Traditional Chinese medicine)	5. 秘方 (Self medication)	6. 飲食控制 (Diet control)	1. 每天用藥 (Daily)	2. 不舒服才用藥 (When I feel uncomfortable)	3. 想到才用藥 (When I feel like taking)	4. 只有飲食控制 (Only through diet)	5. 什麼都沒做 (None)
1.糖尿病(Diabetes)	0	1	___年 (Years)	0	1	2	3	4	5	6	1	2	3	4	5
2.高血壓(Hypertension)			___年 (Years)												
3.心臟病(Heart disease)			___年 (Years)												
4.B 型肝炎 (Hepatitis B)			___年 (Years)												
5.C 型肝炎(Hepatitis C)			___年 (Years)												
6.肝硬化(Liver cirrhosis)			___年 (Years)												
7.腎臟病 (Kidney disease)			___年 (Years)												
8.痛風 (Gout)			___年 (Years)												
9.高血脂 (Hyperlipidemia)			___年 (Years)												

10.中風 (Stroke)	___年 (Years)
11.骨質疏鬆 (Osteoporosis)	___年 (Years)
12.結核病 (Tuberculosis)	___年 (Years)
13.精神疾病(Mental illness)	___年 (Years)
14.氣喘 (Asthma)	___年 (Years)
15. 慢性潰瘍結腸炎 (Chronic ulcer colitis)	___年 (Years)
16. 其他神經疾病 (Other neurological diseases)	___年 (Years)
17.家族性大腸瘜肉症 (Familial adenomatous polyposis)	___年 (Years)
18.遺傳性非瘜肉大腸直腸癌 (Hereditary nonpolyposis colorectal cancer)	___年 (Years)
19.其他癌症 (Other cancers)	___年 (Years)
20.其他 (Other diseases)	___年 (Years)

第五部份：用藥、保健食品習慣(Part 5: The habit of using medicines and supplements)

請問您平常是否有在使用或服用藥品或保健食品？(Do you usually use or take medicines or supplement?)

種類(Type)	服用原因 (Reason for taking)			服用頻率 (Frequency of taking)				
	1. 治療 Treatment	2. 保健 Prevention	3. 皆有 Both	1. 每週七次以上 7 times or more/ week	2. 每週五－六次 5-6 times / week	3. 每週三－四次 3-4 times / week	4. 每週一－二次 1-2 times / week	5. 很少吃或不吃 Few or Never
1.提神的藥 (Energy pills)	1	2	3	1	2	3	4	5
2.安眠藥 (Sleeping pills)	1	2	3	1	2	3	4	5
3.鎮靜的藥 (Antidepressants)	1	2	3	1	2	3	4	5
4.止痛藥 (Painkiller)	1	2	3	1	2	3	4	5
5.減肥藥 (Weight loss pills)	1	2	3	1	2	3	4	5
6.中藥 (Traditional Chinese medicine)	1	2	3	1	2	3	4	5
7.維他命 A(Vitamin A)	1	2	3	1	2	3	4	5
8.維他命 B 群(Vitamin B)	1	2	3	1	2	3	4	5

9.維他命 C (Vitamin C)	1	2	3	1	2	3	4	5
10.維他命 D (Vitamin D)	1	2	3	1	2	3	4	5
11.維他命 E (Vitamin E)	1	2	3	1	2	3	4	5
12.綜合維他命 (Synthetic vitamin)	1	2	3	1	2	3	4	5
13.鈣片(Calcium)	1	2	3	1	2	3	4	5
14.動物類產品，如深海魚油、甲殼質 (Animal products e.g., fish oil, chitin)	1	2	3	1	2	3	4	5
15.本草植物，如靈芝、冬蟲夏草 (Herbal plants, e.g., Ganoderma lucidum, Cordyceps sinensis)	1	2	3	1	2	3	4	5
16.特定營養素，如蛋白質、卵磷脂 (Specific nutrients e.g., protein, lecithin)	1	2	3	1	2	3	4	5
17.酵素和益菌類，如啤酒酵母和優酪乳 (Enzymes and probiotics e.g., brewer's yeast and yogurt)	1	2	3	1	2	3	4	5
18.生化產品，生化技術萃取如蜂王乳、蒜精、蘋果醋 (Biochemical products and extraction e.g., royal jelly, garlic, apple cider vinegar)	1	2	3	1	2	3	4	5
19.其他 (Others)__________________	1	2	3	1	2	3	4	5

第六部份：女性以往經歷 (Part 6: Women's past experiences) (僅問女性)(only women)

1.月經史 (Menstrual history) ：初經年齡 (Menarche age): ____歲 (實歲) (year), □99 不清楚(忘記了) (not sure)

是否規則？(Is your menstrual cycle regular?)

0 否(No)

1 是(Yes), 平均_______天月經來潮一次 (Menstrual cycle __days)

是否已停經？(Menopause?)

0 否(No)

1 是(Yes), 停經原因(reason)：

1 **卵巢切除**(Ovariectomy)；**年齡** (age)_______**歲(實歲)**

2 子宮切除(Hysterectomy)；年齡 (age)_______歲(實歲)

3 **自然停經**(Natural menstruation)；**年齡** (age)_______**歲(實歲)**

4 其他(Others)：____________；年齡(age)_______歲(實歲)

2.用藥史：是否服用荷爾蒙補充劑？(Medication history: Do you take hormone supplements) 0 否 (No) 1 是 (Yes)；_______歲開始 (Age at which you began taking)服用 (How many years)_______年使用原因 (Reason)：_____________________

是否服用口服避孕藥？(Do you take oral contraceptives?) 0 否(No) 1 是 (Yes)；_______歲開始 (Age at which you began taking) 服用 (How many years)_______年使用原因 (Reason)：___________

3.懷孕次數 (Pregnancies)： □ □ □ □ □ □ □ □ □ □) 次(Number of times)

生產次數 (Parity)： □ □ □ □ □ □ □ □ □ □) 次 (Number of times)

流產次數 (Abortions)： □ □ □ □ □ □ □ □ □ □) 次 (Number of times)

生產胎次(Parity)	生產時您的年齡(實歲) (Age at birth)	哺乳方式 (Breastfeeding method)		
		母乳(1) (Breast milk)	奶粉(2) (Milk powder)	混合(3) (Mixed)
第一胎 (First born)	______歲 (Age)	1	2	3
第二胎 (Second born)	______歲 (Age)	1	2	3
第三胎 (Third born)	______歲 (Age)	1	2	3
最後一胎 (Last born)	______歲 (Age)	1	2	3

4.乳房自我檢查結果 (Self-breast examination):

4-1 請問您是否做過乳房自我檢查？ (Have you ever done a self-breast examination?) 2 未做 (No) 1 有做 (Yes) **【續問 4-2】** (Continued)

4-2 最近是否有下列現象之一？(Have you recently had one of the following phenomena?)

1 有 (Yes)：乳房有腫塊、流血、不正常分泌物、皮膚病變或腋下有腫塊 (Breast has a lump, bleeding, abnormal secretions, skin lesions or a lump under the armpit)

0 無 (No)

5.有無接受過醫師或護理人員理學觸診：(Have you ever been palpated by a physician or nursing staff?)

1 有接受過，診斷結果為 (Received the results)： 0 無異常(Normal) 1 有腫塊塊或其他異常(Have a lump and other abnormalities) 2 無接受過 (Did not receive the results)

6.有無得過下列疾病？(Have you ever had the following diseases?)

4 有(Yes)：(1 乳房良性相關疾病 (Breast cancer related diseases) 2 乳癌 (Breast cancer) 3 其他癌症) (Other cancers)

0 無 (No)；

7.家族史：與您有血緣的家屬中，有沒有人得過乳癌？(Family history: Has any of your blood relatives had breast cancer?)

1 有(Yes)(**請填入人數**)(Number of people)(姑姑指父親的親姊妹，阿姨指母親的親姊妹)(Partenal and maternal aunts)

血緣關係 (Relative)	母親 (Mother)	姊妹 (Sister)	女兒 (Daughter)	祖母 (Paternal grandmother)	外祖母 (Maternal grandmother)	姑姑 (Paternal aunt)	阿姨 (Maternal aunt)
罹患乳癌人數 (Number with breast cancer)							

0 無 (No)

8.近三年內是否做過乳房 X 光攝影檢查？(Have you ever undergone mammography in the past three years?)

1 是 (Yes)，最近一次檢查為(Most recent)____年(Year)____月(Month)； 0 否 (No)

第七部份：健康量表 (Part 7: Health Status)

下列問題是有關於**最近兩星期**以來，您對自己的健康狀態的看法，請您根據自己真正的感覺，請選擇最符合您健康狀況的答案並在該欄位上「○」內以塗滿，謝謝！(The following questions are about your opinion concerning your health status in the last two weeks. Please choose the answer that best suits your health status and fill in the field according to your true feelings. Thank you)

1.你是否覺得頭痛或是頭重重漲漲的有壓迫感？(Do you feel headache?)

1 否 (No) 是 (Yes) 2 跟平時差不多 (Mild) 3 比平常時較覺得 (Severe) 4 比平常時更覺得 (Very severe)

2. 你是否覺得心悸或心跳加快擔心可能得了心臟病？(Do you feel that your heartbeat is too fast and you may have a heart attack?)

1 否 (No)是 (Yes) 2 跟平時差不多 (Mild) 3 比平常時較覺得 (Severe) 4 比平常時更覺得 (Very severe)

3. 你是否覺得胸前不適悶悶的有壓迫感？(Do you feel discomfort in your chest?)

1 否 (No)是 (Yes) 2 跟平時差不多 (Mild) 3 比平常時較覺得 (Severe) 4 比平常時更覺得 (Very severe)

4. 你是否覺得手腳發抖或發麻？(Do your hands and feet feel shivery and numb)

1 否 (No)是 (Yes) 2 跟平時差不多 (Mild) 3 比平常時較覺得 (Severe) 4 比平常時更覺得 (Very severe)

5. 你是否覺得睡眠不好？(Do you feel that you don’t sleep well)

1 否 (No)是 (Yes) 2 跟平時差不多(Mild) 3 比平常時較覺得 (Severe) 4 比平常時更覺得 (Very severe)

6. 你是否覺得許多事情對您是負擔？(Do you feel burdened with too many things)

1 否 (No)是 (Yes) 2 跟平時差不多(Mild) 3 比平常時較覺得 (Severe) 4 比平常時更覺得 (Very severe)

7. 你是否和家人相處得來？(Do you get along with your family?)

1 否(No) (和家人相處不好) (I don’t get along with my family)是 (Yes) 2 跟平時差不多(Mild) 3 比平常時較覺得 (Severe) 4 比平常時更覺得 (Very severe)

8. 你是否對自己失去信心？(Are you losing confidence in yourself?)

1 否 (No)是 (Yes) 2 跟平時差不多(Mild) 3 比平常時較覺得 (Severe) 4 比平常時更覺得 (Very severe)

9. 你是否覺得神經兮兮、緊張兮兮？(Do you feel nervous?)

1 否 (No)是 (Yes) 2 跟平時差不多(Mild) 3 比平常時較覺得 (Severe) 4 比平常時更覺得 (Very severe)

10. 你是否對未來充滿希望？(Do you feel hopeful about the future)

1 否 (No) (對未來毫無希望) (I don’t feel hopeful) 是 (Yes) 2 跟平時差不多(Mild) 3 比平常時較覺得 (Severe) 4 比平常時更覺得 (Very severe)

11. 你是否覺家人或親友會令您擔心？(Are you worried about your loved ones)

1 否 (No)是 (Yes) 2 跟平時差不多(Mild) 3 比平常時較覺得 (Severe) 4 比平常時更覺得 (Very severe)

12. 你是否覺得對生活毫無希望？(Do you feel hopeless about life?)

1 否 (No)是 (Yes) 2 跟平時差不多(Mild) 3 比平常時較覺得 (Severe) 4 比平常時更覺得 (Very severe)

第八部份：SF-36 生活品質問卷 (Part 8: SF-36 Quality of Life)

本調查的目的在探討您對自己健康的看法。這些資訊將能幫助您記錄您的感受，以及您在執行日常生活的能力 (The purpose of this survey is to explore your views about your health. This information will help you to record your feelings and your ability to perform your daily activities)。

請回答下列各問題並圈選依適當答案。如您對某一問題的回答不能確定，還是請您盡可能選一個最適合的答案。在本部分所指**過去一個月內**，係指**從今天往前算三十天內** (Please answer the following questions and circle the appropriate answers. If you are not certain about your choice, please select the best option. The past month referred to in this section means within the past 30 days)

1.一般來說，您認為您目前的健康狀況是 (Generally speaking, what do you think about your current health status)

(請僅塗滿一項答案) (Please choose only one answer)

極好的 (Excellent) 1

很好(Very good).. 2

好(Good).. 3

普通(Average) 4

不好(Not good).. 5

2.和前一年前比較，您認為您目前的健康狀況是？(Compared with the previous year, what do you think about your current health status)

(請僅塗滿一項答案) (Please choose only one answer)

比一年前好很多 (Much better) 1

比一年前好一些 (Better)... 2

和一年前差不多 (Almost the same)... 3

比一年前差一些 (Worse) 4

比一年前差很多 (Worst)... 5

3.下面是一些您日常可能從事的活動，請問您目前健康狀況會不會限制您從事這些活動？如果會，到底限制有多少？(Here are some of the activities you might do on a daily basis. Will your current health status restrict you from engaging in these activities? If so, how many restrictions are there?)
(每行請僅塗滿一項答案) (Please choose only one answer)

活動 (Activity)	會，受到很多限制 (Yes, many restrictions)	會，受到一些限制 (Yes, some restrictions)	不會，完全不受限制 (No, no restrictions)
a.費力活動，例如跑步、提重物、餐與激烈運動 (Laborious activities such as running, lifting heavy objects, and intense sports)	1	2	3
b.中等程度活動，例如搬桌子、拖地板、打保齡球、或打太極拳 (Moderate activities such as moving tables, mopping the floor, bowling, or playing Tai Chi)	1	2	3
c.提起或攜帶食品雜貨 (Lift or carry groceries)	1	2	3
d.爬數層樓梯 (Climb several staircases)	1	2	3
e.爬一層樓梯 (Climb a staircase)	1	2	3
f.彎腰、跪下或蹲下(Stoop, kneel and squat)	1	2	3
g.走路超過 1 公里 (Walk for more than 1 Km)	1	2	3
h.走路數個街口 (Walk through a few streets)	1	2	3
i.走過一個街口 (Walk through one street)	1	2	3
j.自己洗澡或穿衣 (Bathe or dress yourself)	1	2	3

4.在**過去一個月內**，您是否曾**因為身體健康問題**，而在工作上或其他日常活動方面有下列任何問題？
(Have you had any of the following problems with your work or other daily activities because your health problems in the past month?)

(每行請僅塗滿一項答案) (Please choose only one answer)

	是 (Yes)	否 (No)
a.做工作或其他活動的時間減少 (Spent less time doing work or other activities)	1	2
b.完成的工作量比您想要完成的較少 (The amount of work done is less than what you want to accomplish)	1	2
c.可以做的工作或是其他活動的種類受到限制 (The work or other activities that you can do are limited)	1	2
d.做工作或其他活動有困難(例如，須更吃力) (Difficulties in doing work or other activities e.g., requires more efforts)	1	2

5.在**過去一個月內**，您是否曾**因為情緒問題**(例如，感覺沮喪或焦慮)，而在工作上或其他日常活動方面有下列任何問題？(Have you had any of the following problems with your work or other daily activities because of emotional problems e.g., feeling depressed or anxious in the past month)?

(每行請塗滿選一項答案) (Please choose only one answer)

	是(Yes)	否(No)
a.做工作或其他活動的時間減少 (Spent less time doing work or other activities)	1	2
b.完成的工作量比您想要完成的較少 (The amount of work done is less than what you want to accomplish)	1	2
c.做工作或其他活動時不如以往小心 (Not as careful as before when working or doing other activities ?)	1	2

6.在**過去一個月內**，您的身體健康或情緒問題，對您與家人或是朋友、鄰居、社團間的平常活動的妨礙程度如何？(How much has your physical health or emotional problems hindered your normal activities with your family, friends, neighbors, and communities in the past month?)

(請僅塗滿一項答案) ((Please choose only one answer)

完全沒有妨礙 (Not at all)........ 1

有一點妨礙 (A little) 2

中度妨礙 (Moderately) 3

相當妨礙 (Considerably) 4

妨礙到極點(Extremely)........ 5

7.在**過去一個月內**，您的**身體**疼痛程度有多嚴重？(How serious has your body pain been in the past month?)

(請僅塗滿一項答案) (Please choose only one answer)

完全不痛 (Not at all)........ 1

非常輕微的痛 (Very mild) 2

輕微的痛 (Mild)........ 3

中度的痛 (Moderate) 4

嚴重的痛 (Severe)........ 5

非常嚴重的痛 (Very severe) 6

7-1 如果會痛，疼痛原因為何？ (If you feel pain, what causes it?)________________________

8.在**過去一個月內**，您的**身體**疼痛對您的日常工作(包括上班及家務)妨礙程度如何？ (How much has

your physical pain been hindering your daily activities, including work and housework in the past month?)

(請僅塗滿一項答案) (Please choose only one answer)

完全沒有妨礙 (Not at all)........................	1
有一點妨礙 (A little)	2
中度妨礙 (Moderately)	3
相當妨礙 (Considerably)	4
妨礙到極點(Extremely)........................	5

9.下列各項問題是關於**過去一個月內**您的感覺及您對週遭生活的感受, 請針對每一問題選一最接近您感覺的答案。在**過去一個月**中有多少時候…… (The following questions are about your feelings in the past month and how you feel about life around you. Please choose the answer that is closest to your feelings for each question. How many times?)

(每行請僅塗滿一項答案) (Please choose only one answer)

	一直都是 (Always)	大部分時間 (Most of the time)	經常 (Usually)	有時 (Sometimes)	很少 (Seldom)	從不 (Never)
a.您覺得充滿活力 (Full of enery)	1	2	3	4	5	6
b.您是一個非常緊張的人 (Very nervous)	1	2	3	4	5	6
c.您覺得非常沮喪, 沒有任何事情可以讓您高興起來？(Do you feel very frustrated and nothing makes you happy?)	1	2	3	4	5	6
d.您覺得心情平靜？(Do you feel calm)	1	2	3	4	5	6
e.您精力充沛？(Do you feel active)	1	2	3	4	5	6
f.您覺得悶悶不樂和憂鬱？(Do you feel depressed?)	1	2	3	4	5	6
g.您覺得筋疲力竭(Do you feel exhausted?)	1	2	3	4	5	6
h.您是一個快樂的人？(Are you a happy person)	1	2	3	4	5	6
i.您覺得累？(Do you feel tired?)	1	2	3	4	5	6

10.在**過去一個月內**, 您的**身體健康或情緒問題**有多少時候會妨礙您的社交活動(如拜訪親友等)？(How many times has your physical health or emotional problems hindered your social activities such as visiting relatives and friends in the past month)

(請僅塗滿一項答案) (Please choose only one answer)

一直都會 (Always) 1

大部分時間會 (Most of the time) 2

有時候會 (Sometimes) 3

很少會 (Seldom) 4

從不會 (Never) 5

11.下列**各個**陳述對您來說有多正確？(How correct are the following statements about you?)

(每行請僅塗滿一項答案) (Please choose only one answer)

	完全正確 (Completely correct)	大部分正確 (Almost correct)	不知道 (I do not know)	大部分不正確 (Almost incorrect)	完全不正確 (Completely incorrect)
a.我好像比別人較容易生病 (I seem more sick than others)	1	2	3	4	5
b.和任何一個我認識的人比起來，我和他們一樣健康 (I am as healthy as other people I know)	1	2	3	4	5
c.我想我的健康會越來越壞 (I think my health may get worse)	1	2	3	4	5
d.我的健康狀況好的很 (My health is very good)	1	2	3	4	5

第九部份：基本資料及意見信箱(Part 9: Basic information)

1.教育程度 (Educational level) ：

1 研究所或以上 (Masters or above) 2 大學(專)(University/College)

3 高中(職) (Senior high school) 4 國(初)中(Junior high school)

5 小學(Elementary school) 6 識字(Literate) 7 不識字 (Illiterate)

2.您的血型: (Blood type)

1 A 型 (Type A) 2 B 型 (Type B) 3 O 型 ((Type O) 4 AB 型 (Type AB) 5 不知道 (Unknown)

3.請問您的父親屬於何種族群？(What ethnic group does your father belong to?)

1 本省客家人 (Hakka) 2 本省閩南人 (Minnan) 3 大陸各省市 (Mainland) 4 原住民 (Aboriginal) 5 其他 (Others)_______

4.請問您的母親屬於何種族群？(What ethnic group does your mother belong to?)

1 本省客家人 (Hakka) 2 本省閩南人 (Minnan) 3 大陸各省市 (Mainland) 4 原住民 (Aboriginal) 5 其他 (Others)_______

5.婚姻狀況: (Marital status)

1 未婚 (Unmarried) 2 已婚 (Married) 3 分居(Separated) 4 離婚(Divorced) 5 鰥寡 (Widowed) 6 其他 (Others)：___________________________________

6.個人年收入 (Personal annual income)

1 30 萬以下(Less than NTD 300,000) 2 30-50 萬 (NTD 300,000-500,000) 3 50-75 萬 (NTD 500,000-750,000) 4 75-100 萬 (NTD 750,000-1,000,000) 5 100-180 萬(NTD1,000,000-1,800,000) 6 180 萬以上 (NTD 1,800,000 and above)

7.現在行業別 (Current industry)

1 「農、林、漁、牧業」(Agriculture, forestry, fishing, animal husbandry) 2 「礦業及土石採取業」(Mining or mining-related) 3 「製造業」(Manufacturing)

4 「水電燃氣業」(Hydropower and gas) 5 「營造業」(Construction) 6 「批發及零售業」(Wholesale and retail)7 「住宿及餐飲業」(Housing and food) 8 「運輸、倉儲及通信業」(Transportation, warehousing and communication) 9 「金融及保險業」(Finance and insurance) 「不動產及租賃業」(Real estate and leasing) ! 「專業、科學及技術服務業」(Professional, scientific and technical services) @ 「教育服務業」(Educational services)

「醫療保健及社會福利服務業 (Healthcare and social welfare services) $ 「文化、運動及休閒服務業」(Culture, sports and leisure services) % 「其他服務業」(Others) ^ 「公共行政業」(Public administration) & 無 (None)

8.現在職業別 (Current occupation)

1 現役軍人 (Military officer) 2 民意代表、行政主管、企業主管及經理人 (Public opinion representatives, executives, business executives and managers) 3 專業人員 (Professional) 4 技術員及助理專業人員(Technician and assistant professional) 5 事務工作人員(General affairs staff) 6 服務工作人員及售貨員(Service staff and salesperson) 7 農、林、漁、牧工作人員 (Agriculture, forestry, fishing, animal husbandry staff) 8 技術工及有關工作人員(Technical workers and related staff) 9 機械設備操作工及組裝工(Mechanical equipment operator and assembler)) 非技術工及體力工 (Unskilled and manual workers) ! 無 (None)

9.過去行業別？(Previous industry)

1 「農、林、漁、牧業」(Agriculture, forestry, fishing, animal husbandry) 2 「礦業及土石採取業」(Mining or mining-related) 3 「製造業」(Manufacturing)

4 「水電燃氣業」(Hydropower and gas) 5 「營造業」(Construction)

6 「批發及零售業」(Wholesale and retail)7 「住宿及餐飲業」(Housing and food) 8 「運輸、倉儲及通信業」(Transportation, warehousing and communication) 9 「金融及保險業」(Finance and insurance) 「不動產及租賃業」(Real estate and leasing) ! 「專業、科學及技術服務業」(Professional, scientific and technical services) @ 「教育服務業」(Educational services)

「醫療保健及社會福利服務業 (Healthcare and social welfare services) $ 「文化、運動及休閒服務業」(Culture, sports and leisure services) % 「其他服務業」(Others) ^ 「公共行政業」(Public administration) & 無 (None)

10.過去職業別？(Previous occupation)

1 現役軍人 (Military officer) 2 民意代表、行政主管、企業主管及經理人 (Public opinion representatives, executives, business executives and managers) 3 專業人員 (Professional) 4 技術員及助理專業人員(Technician and assistant professional) 5 事務工作人員(General affairs staff) 6 服務工作人員及售貨員(Service staff and salesperson) 7 農、林、漁、牧工作人員 (Agriculture, forestry, fishing, animal husbandry staff) 8 技術工及有關工作人員(Technical workers and related staff) 9 機械設備操作工及組裝工(Mechanical equipment operator and assembler)) 非技術工及體力工 (Unskilled and manual workers) ! 無 (None)

11.出生地：(Place of birth)

1 台灣以外地區 (Outside of Taiwan)：__________

2 台灣_(Taiwan)______縣(市) (County, city)________(鄉鎮市) (Township)

12.個人遷徙狀況, 以跨縣市來計算, 從出生到目前為止共_____次(例如至他縣求學、就業等等) (Personal migration status, determined by inter-county and city movements from birth to date e.g for studies, employment, etc.)

	居住地點 (Place of residence)	約居住幾年 (Duration of stay)	原因(如個人/關係人就學、個人/關係人就業、結婚…. (Reasons e.g., personal/relationships, employment, marriage...)
第一次居住地 (First place of residence)	________縣(市) (County)________ (鄉鎮市) (Township)	_________年 (Years)	
第二次居住地 (Second place of residence)	________縣(市) (County)________ (鄉鎮市) (Township)	_________年 (Years)	
第三次居住地 (Third place of residence))	________縣(市) (County)________ (鄉鎮市) (Township)	_________年 (Years)	
第四次居住地 (Fourth place of residence)	________縣(市) (County)________ (鄉鎮市) (Township)	_________年 (Years)	
第五次居住地 (Fifth place of residence)	________縣(市) (County)________ (鄉鎮市) (Township)	_________年 (Years)	
第六次居住地 (Sixth place of residence)	________縣(市) (County)________ (鄉鎮市) (Township)	_________年 (Years)	

13. 請問現在您**戶口名簿**上記載共有_____人(How many people are contained in your household registry?__；現在和您**住在一起**共有_____人(How many people are you currently living with?___

14.請問您怎麼知道今天這項活動的？(可複選) (How did you know about this program?) (Multiple answers are accepted)

1 電話(Telephone) 2 簡訊(Text message) 3 信件(Mail) 4 工作人員拜訪(Staff visit) 5 親友、鄰居邀約(Friends and relatives) 6 其他(Others):_______

15.您對於今天的服務感到滿意嗎？(Are you satisfied with today's interview?)

1 非常滿意(Very satisfied) 2 滿意(Satisfied) 3 普通(Neutral) 4 不滿意(Dissatisfied) 5 非常不滿意(Very dissatisfied) 滿意或不滿意的原因？(Reason for satisfaction or dissatisfaction)______

訪問結束時間：______時______分 (End of interview)：______Hour______Minute)

以上問卷到此結束，感謝您的協助　謝謝！(The above questionnaire ends here. We are grateful for your cooperation, and would like to thank you once again.)
